# Humans versus AI: whether and why we prefer human-created compared to AI-created artwork

**DOI:** 10.1186/s41235-023-00499-6

**Published:** 2023-07-04

**Authors:** Lucas Bellaiche, Rohin Shahi, Martin Harry Turpin, Anya Ragnhildstveit, Shawn Sprockett, Nathaniel Barr, Alexander Christensen, Paul Seli

**Affiliations:** 1 Department of Psychology and Neuroscience, Duke University, 417 Chapel Drive, Durham, NC 27708 USA; 2 Department of Psychology, University of Waterloo, Waterloo, ON Canada; 3 Department of Psychiatry, University of Cambridge, Cambridge, UK; 4 MDes in Interaction Design Program, California College of the Arts, San Francisco, CA USA; 5 School of Humanities and Creativity, Sheridan College, Oakville, ON Canada; 6 Psychology and Human Development, Peabody College, Vanderbilt University, Nashville, TN USA

**Keywords:** Aesthetics, Artificial intelligence, Visual art, Creativity, Judgements

## Abstract

With the recent proliferation of advanced artificial intelligence (AI) models capable of mimicking human artworks, AI creations might soon replace products of human creativity, although skeptics argue that this outcome is unlikely. One possible reason this may be unlikely is that, independent of the physical properties of art, we place great value on the imbuement of the human experience in art. An interesting question, then, is whether and why people might prefer human-compared to AI-created artworks. To explore these questions, we manipulated the purported creator of pieces of art by randomly assigning a “Human-created” or “AI-created” label to paintings actually created by AI, and then assessed participants’ judgements of the artworks across four rating criteria (Liking, Beauty, Profundity, and Worth). Study 1 found increased positive judgements for human- compared to AI-labelled art across all criteria. Study 2 aimed to replicate and extend Study 1 with additional ratings (Emotion, Story, Meaningful, Effort, and Time to create) intended to elucidate *why* people more-positively appraise Human-labelled artworks. The main findings from Study 1 were replicated, with narrativity (Story) and perceived effort behind artworks (Effort) moderating the label effects (“Human-created” vs. “AI-created”), but only for the sensory-level judgements (Liking, Beauty). Positive personal attitudes toward AI moderated label effects for more-communicative judgements (Profundity, Worth). These studies demonstrate that people tend to be negatively biased against AI-created artworks relative to purportedly human-created artwork, and suggest that knowledge of human engagement in the artistic process contributes positively to appraisals of art.

**Supplementary Information:**

The online version contains supplementary material available at 10.1186/s41235-023-00499-6.

## Introduction

Art is widely considered to be a uniquely human phenomenon. It can encapsulate and communicate our emotions, be used to express our individualistic and communal experiences, and serve as a social commentary on these experiences, all of which are elements that are commonly thought to be human-specific (Chatterjee, 2014). Yet, art is also a perceptual product that is engaged through the senses, sometimes independently of the experient’s awareness of the inclusion (or lack thereof) of these human elements in artworks they evaluate. Here, an important distinction emerges between art as (1) a *purely physical stimulus* and (2) a *deeper communicative medium* of the human experience. The importance of this distinction lies in its ability to permit investigations of the separate and joint influences that these two conceptions of art may have on people’s appraisals of art, which in turn contributes to our understanding of the factors and processes involved in aesthetic appraisals. And, with the recent development of highly advanced artificial intelligence (AI) models that can produce, as *purely physical stimuli*, high-quality artworks that are indiscernible from human-created artworks (Gangadharbatla, 2022; Johnson, 1997; Mazzone & Elgammal, 2019), the Computer Age in which we live has afforded considerable experimental control over these two conceptions of art. Here, across two studies, we explored the question of whether and why humans might tend to prefer ostensibly human-created artworks over AI-created artworks.

Although the process of appraising artworks undoubtedly involves a subjective element, consistent preferential patterns have nevertheless emerged across peoples’ aesthetic judgements of such artworks (e.g., blue is the most preferred color, whereas yellow is most disliked [Bornstein, 1975; Komar & Melamid, 1999; Palmer et al., 2013]; and representational art is enjoyed over abstract art [Heinrichs & Cupchik, 1985; Kettlewell et al., 1990; Knapp & Wulff, 1963; Mastandrea et al., 2011, 2021]). However, the ways in which we judge art are often sensitive to factors in the environment, not just intrinsic preferences. One common finding across literatures of judgement and decision-making is that labels play an important role in people’s general evaluations of things. For instance, people prefer wine they are falsely told is more expensive than other wine (Plassmann et al., 2008). The influence that a mere label has on our judgements is also made evident by research showing that, when participants are provided Coca-Cola either with or without its label, they report greater enjoyment of the labelled beverage than the non-labelled beverage, despite their otherwise identical compositions (McClure et al., 2004). As demonstrated by these studies, our judgements rely heavily on contextual information. And, perhaps unsurprisingly, the role that context plays in our appraisals extends to judgements of art as well. For example, when Newman and Bloom (2012) provided participants artworks labelled either as originals or as identical forgeries, participants preferred the originals over the forgeries: a finding that was taken to indicate that humans are sensitive to an authentic process of creative production as determined simply by a label. In other words, people consider non-sensory aspects of art, like context and background, in their judgements and evaluations of art (Blank et al., 1984; Chatterjee & Vartanian, 2016; Winner, 1982).

The influential role that labels play in our judgements is also made clear in contemporary lines of research focused on AI. The general finding emerging from this research is that, in many instances, the label of “AI” is taken to be a pejorative. For instance, Liu et al. (2022) conducted an experiment wherein participants were asked to read a number of emails and were told that some of the emails (but not others) were drafted with the help of an AI-based language model. Results indicated that, when participants were told that AI played a role in drafting the email, their reports of trust in the email writer decreased. This anti-AI bias seems especially apparent in domains that people assume to be human-specific (e.g., those concerning affect or creativity; Wilson, 2011). Research has only just begun to connect the spheres of aesthetics and AI, yet, the discussion of AI-produced creativity arose many decades ago. Indeed, in his 1950 Presidential Address to the American Psychological Association (APA), JP Guilford foreshadowed the development of AI in modern society, noting that, if advances in “thinking machines” (i.e., AI) reached their forecasted height, then “the only economic value of brains left would be the creative thinking of which they are capable” (Guilford, 1950). While Guildford’s position is certainly provocative, he may have overestimated the extent to which the apparent ‘last bastion’ of human utility— “creativity”—is uniquely human; instead, it could be that, with recent advances in highly advanced “thinking machines,” even our creative abilities could lose their economic value when competing with the abilities of AI.

Initial forays into the AI-art interaction seem to suggest that, not only is AI art sufficiently sensorily similar to human-created art that people fail to accurately discern its true creator (human or AI), but also that people tend to derogate AI-created art as compared to human-created art (Chamberlain et al., 2018; Gangadharbatla, 2022; Mazzone & Elgammal, 2019). For example, Chamberlain et al. (2018) found that participants rated human-created artworks as higher in aesthetic value than AI-created artworks. Interestingly, however, this bias was malleable: indeed, when participants were shown videos of robots that were physically producing art, participants’ anti-AI art judgements were reduced, suggesting that people may consider the level of artistic effort exerted by the creator as a marker of the quality of the produced artwork. Similar findings emerged in a more-recent study in which participants were shown, and asked to provide judgements of, two abstract paintings (Chiarella et al., 2022). While both paintings were in fact created by humans, by randomly assigning a label of “human-created” or “AI-created” to each painting, the researchers deceived participants into believing that one of the paintings was created by AI. Results of this study indicated that participants tended to prefer the painting that was labelled “human-created” relative to the painting labelled “AI-created.” Notably, this preference for human over AI art is not specific to visual art. In fact, several recent studies have reported similar anti-AI findings in music (Shank et al., 2022), creative writing (Raj et al., 2023), dance (Darda & Cross, 2023), poetry (Köbis & Mossink, 2020), and non-art texts (Darda et al., 2023). This bias in aesthetic judgement is further intensified when judging more “human” aspects of art, like evoked emotion, suggesting the need for multiple judgement criteria in AI aesthetics research (Raj et al., 2023).

Critically, however, other studies have yielded results that are at odds with those from the aforementioned studies, finding little-to-no differences in evaluations of human- and AI-created artworks (Hong & Curran, 2019; Israfilzade, 2020; Xu et al., 2020). Additionally, though not an explicit investigation of aesthetic-judgements, per se, one study found that participants were equally likely to consider hypothetical products created by human and AI artists as “art” (Mikalonytė & Kneer, 2022). Put differently, people did not tend to perceive AI artists’ hypothetical products as being lesser than human artists’ hypothetical products. Given these mixed results, it is challenging to ascertain whether humans do in fact prefer human-created artworks over AI-created artworks, and, if so, why this is so. Thus, here, we examined this question with the intention of shedding more light on their answers.

### The current studies

Across two studies, we sought to extend the extant—albeit sparse—literature on attitudes toward artworks created by humans as compared to artworks created by AI platforms. Our studies had three primary aims. First, while a handful of studies have examined the question of whether people differ with respect to their judgements of human-created versus AI-created artworks, at noted above, mixed results have been yielded, leaving the answer to this question unclear. Thus, by employing two large-sample (*Ns* = 150 and 151), highly powered, within-subjects studies including relatively large stimulus sets, we sought to provide greater clarity on the answer to this question. We hypothesized that humans will indeed show a preference for human-labelled art relative to AI-labelled art.

Second, we sought to identify some of the more-complex criteria that people might rely upon when making aesthetic judgements. Many aesthetic studies have used simple probes assessing general “liking” and “aesthetic beauty” to index art judgements (for a review, see Chatterjee & Cardillo, 2022). However, most models of aesthetics consider aesthetic judgements to be a multifaceted and/or hierarchical cascade of evaluations, implicating more-complex and elaborative processes in judgements of art that seek to understand meaning and communication through the piece (Berlyne, 1960, 1971; Chatterjee & Vartanian, 2016; Cupchik & Berlyn, 1979; Graf & Landwehr, 2015; Leder et al., 2004; Silvia, 2005). This is supported, in part, by the growing discussion of art not only as a bottom-up appraisal of only visual features that induces liking or judgements of beauty, but a device for communication and socio-epistemic value (see Sherman & Morrissey, 2017). This distinction considers human expression and, as such, is essential to investigate in aesthetic judgements of art created by AI. Thus, in Study 1, in addition to simply indexing Liking and Beauty, which we consider to be more-passive, surface-level appraisals in line with fluency models of aesthetics (see Leder et al., 2004; Graf & Landwehr, 2015), we also obtained assessments of how profound participants found each painting to be, and how much money they would (hypothetically) spend on each painting. These latter criteria require higher cognitive elaboration to determine communicative properties of art, as discussed in Graf and Landwehr (2015) and Sherman and Morrissey (2017). In Study 2, we further developed our probing procedure by, in addition to asking the four questions from Study 1 (i.e., Liking, Beauty, Profundity, Worth), asking participants to rate each painting on the following additional communicative attributes of the art to understand potential interactions between surface-level and communicative engagement processes: emotionality (how much the artwork evoked an emotion in the viewer; Emotion), narrativity (the degree to which the artwork portrayed an imagined narrative in the viewer; Story), meaningfulness (how personally meaningful the artwork was to the viewer; Meaningful), perceived effort (how much effort the viewer thought went into the creation of the artwork; Effort), and perceived time (how much time the viewer thought went into the creation of the artwork; Time). We predicted that the anticipated preference for human-labelled art over AI-labelled art can be, at least partially, explained by a perceived lack of integration of the human experience in AI-created artworks as measured by these wide-ranging aesthetic criteria.

Third, given that aesthetic appraisals are personal and subjective (Chatterjee, 2014; Roseman & Evdokas, 2004), also imperative in their investigation is consideration of individual differences that might reliably predict these judgements. In this vein, past work has considered the personality trait of openness to experience and its interactions with artwork and other creative outputs (Kaufman, 2013; McCrae, 2007; McCrae & Greenberg, 2014), with Silvia et al. (2015) considering it “an essentially aesthetic trait” (p. 376). In addition, openness has been shown to influence judgements of art depending on painting type, such that people who are more open to experience tend to have an increased appreciation for abstract art compared to less-open participants (Feist & Brady, 2004). Other personality traits explored in aesthetics and creativity research include empathy and embodied cognitions (Freedberg & Gallese, 2007; Rusu, 2017), and beliefs of creative mindsets (Hass et al., 2016; Karwowski, 2014). To provide greater clarity on the possible influence of individual-differences measures on appraisals of artwork, in Study 1, we assessed age and scores on the cognitive reflection test (CRT), and in Study 2, age, CRT scores, openness to experience, personal attitudes toward AI, empathy, growth mindsets, and fixed mindsets (below, we outline our rationale for inclusion of these individual-differences measures).

## Study 1

### Method

Study 1 was approved by the University of Waterloo Research Ethics Board (31067). All data and stimuli can be found at https://osf.io/cgw8v/.

#### Participants

One-hundred and fifty participants, each with at least 100 approved human intelligence tasks and an approval rating of above 90%, were recruited through Amazon’s Mechanical Turk. Power analyses were not performed a priori. Instead, we reviewed the most closely related studies we could find, finding that, in Study 1 of Chamberlain et al. (2018), 65 participants completed the study. To ensure our study was well-powered, we decided to nearly triple this sample size. Participants were told at sign-up that they would be evaluating pieces of art and filling out questionnaires meant to probe the way they think. Participants were directed to Qualtrics to complete the study, and then were debriefed following completion of the study and were compensated for their time. One participant was excluded for bad data (i.e., they used the same response throughout the entire study), resulting in 149 participants (*M*_age_ = 42.35, *SD* = 11.59; female = 65).

#### Materials

**Fig. 1:**
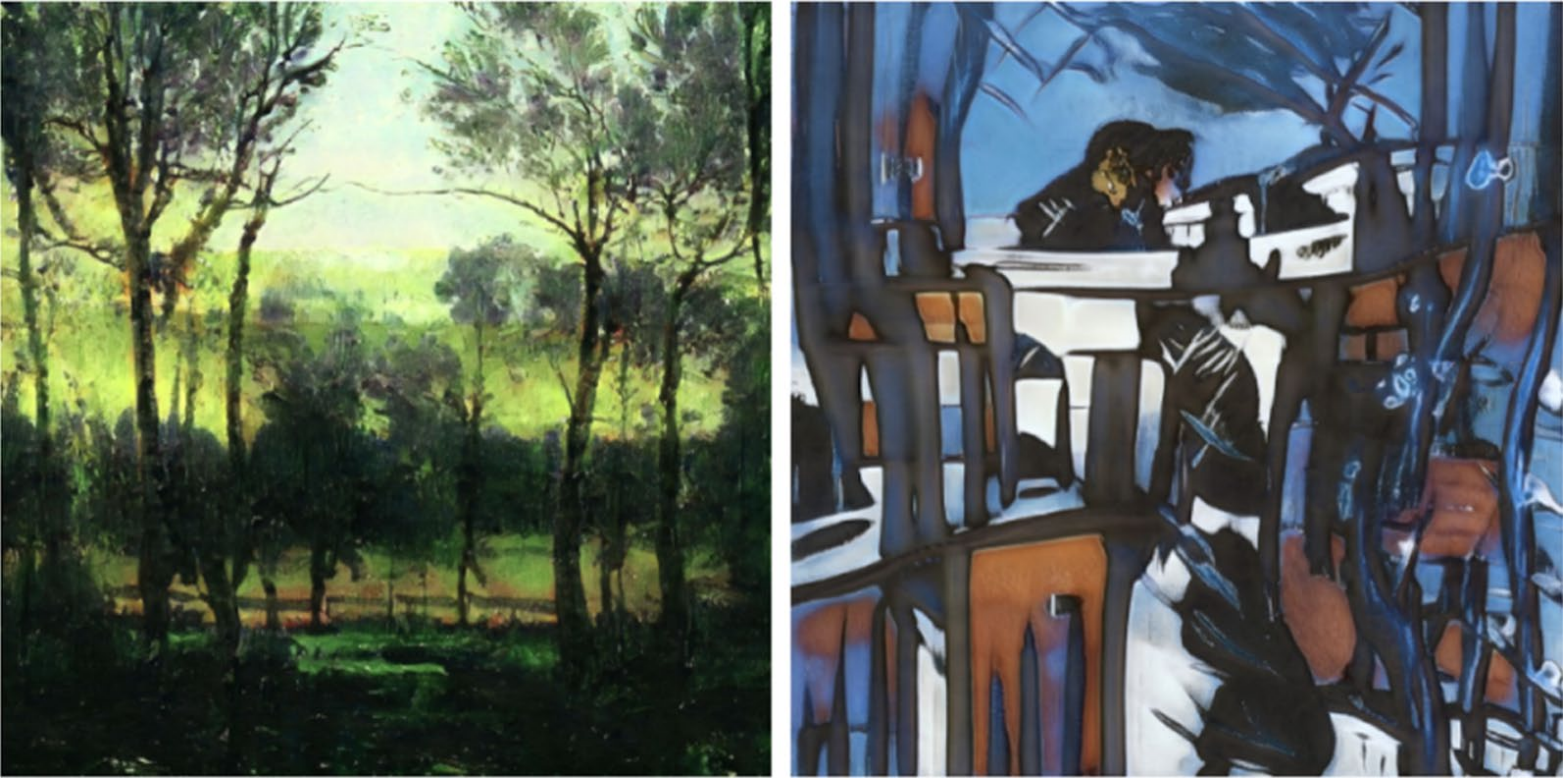
Sample images (representational and abstract, respectively) taken from Artbreeder. See https://osf.io/cgw8v/ for all stimuli

Thirty AI-created paintings, considered by the authors of this article to be of high quality, were taken from ArtBreeder, which is a machine learning website that produces art (see Fig. 1 for sample images). The images were open-source and pre-existing and were not created by the authors. While it might seem most reasonable to obtain artworks from both AI and humans (and not deceive participants), one possible problem with this approach is that there could be error introduced into the selection process. For instance, although one could do their best to ensure that any selected human and AI artworks are comparable, without first conducting a norming study first (across all of our measures), this outcome could not be verified. For this reason, we opted to present a stimulus set that was exclusively AI-created.

Of the 30 AI-created paintings, half (15) were representational (i.e., reflected an easily recognizable figure or object), and half (15) were abstract (i.e., they included partially or completely unrecognizable referents), as rated by the first author. This distinction was made in the present study because past results have repeatedly revealed that people tend to prefer representational over abstract paintings (Heinrichs & Cupchik, 1985; Kettlewell et al., 1990; Knapp & Wulff, 1963; Mastandrea et al., 2011, 2021). Here, we not only wanted to attempt to replicate this finding, but also to determine whether evaluations of different painting types (representational and abstract) differ as a function of whether the purported creator was a human or AI. Research on AI-art perceptions has primarily used abstract paintings as stimuli, which could limit our understanding of the true role of painting type in aesthetic judgements (Chiarella et al., 2022; Israfilzade, 2020). However, Chamberlain et al. (2018) presented varied painting types but found no interaction between painting creator and painting type on general aesthetic value ratings; with our extension of rating criteria, potentially different processes of judgements that may rely both on painting type and creator could be illuminated. Relatedly, Gangadharbatla (2022) found an increased willingness for people to believe that abstract art tends to be created by AI (compared to humans), whereas Chamberlain et al. (2018) found an increased willingness for people to believe that representational art tends to be created by humans. Thus, it seems that painting type does bear some relationship to the creator of the painting; to date, however, this is unclear.

Importantly, each image was presented in random order and had individual-level randomization of a label of “human-created” or “AI-created.” In other words, different labels were assigned to different images across participants. Thus, on average, participants were presented 15 images with an AI label, and 15 images with a human label, even though all images were in fact AI-created.^1^ Participants were asked to rate each image on the following criteria: “How much do you like this image?” (Liking), “How beautiful/aesthetically pleasing is this image?” (Beauty), “How profound or meaningful is this image?” (Profundity), and “How much money would this work be worth?” (Worth). All criteria were answered on a 1–5 Likert scale [“Not at all”… “Very much”], except for Worth, which was answered on a 1–5 Likert scale with possible responses [“None at all”… “Worth quite a lot”]. In addition, for each image, participants were asked “Based on the label above, was this image created by a human or an artificial intelligence computer program?” (Label-Check). This question was used as an attention check. Incorrect responses to the Label-Check were excluded from data analysis, resulting in the removal of 113 trials (out of 4470 total trials across the 149 participants). No participants themselves were removed on the basis of this attention check, only trials.

Before rating the images, participants were asked to complete the 7-item cognitive reflection test (CRT; Frederick, 2005; Toplak et al., 2014). This extended CRT, which has been shown to have high internal consistency (*⍺* = 0.74), consists of seven numeracy questions and is administered to assess an individual’s cognitive ability to override instinctual responses for a correct answer to the problem (Campitelli & Gerrans, 2014). Thus, the CRT considers individual differences in quantitative skills and bias-overriding. Our rationale for including the CRT was to determine whether CRT scores predict different ratings across human- and AI-created artwork: If we do observe an anti-AI bias—with more-positive judgements for human artworks than AI artworks—then one possibility is that individuals who have a greater ability to override their intuitions may show less of a bias, given that this bias may be the result of an automatic (intuitive) response to devalue artworks created by AI. Notably, both CRT and age were included as exploratory variables.

#### Procedure

Participants first provided informed consent following a description of the nature of the study. Next, they were given two bot checks (zero participants failed these checks, and all of them therefore moved forward with the study). Participants were then directed to complete demographic information, followed by the CRT. When finished with these tasks, participants received instructions for the remaining portion of the survey, which asked them to rate the 30 AI-created paintings on Liking, Beauty, Profundity, and Worth, with randomized labels of AI- or human-created. Following ratings, participants were debriefed on the purpose of the study, once again provided consent for their data to be used for analyses, and were redirected for compensation.

### Study 1 results

**Fig. 2:**
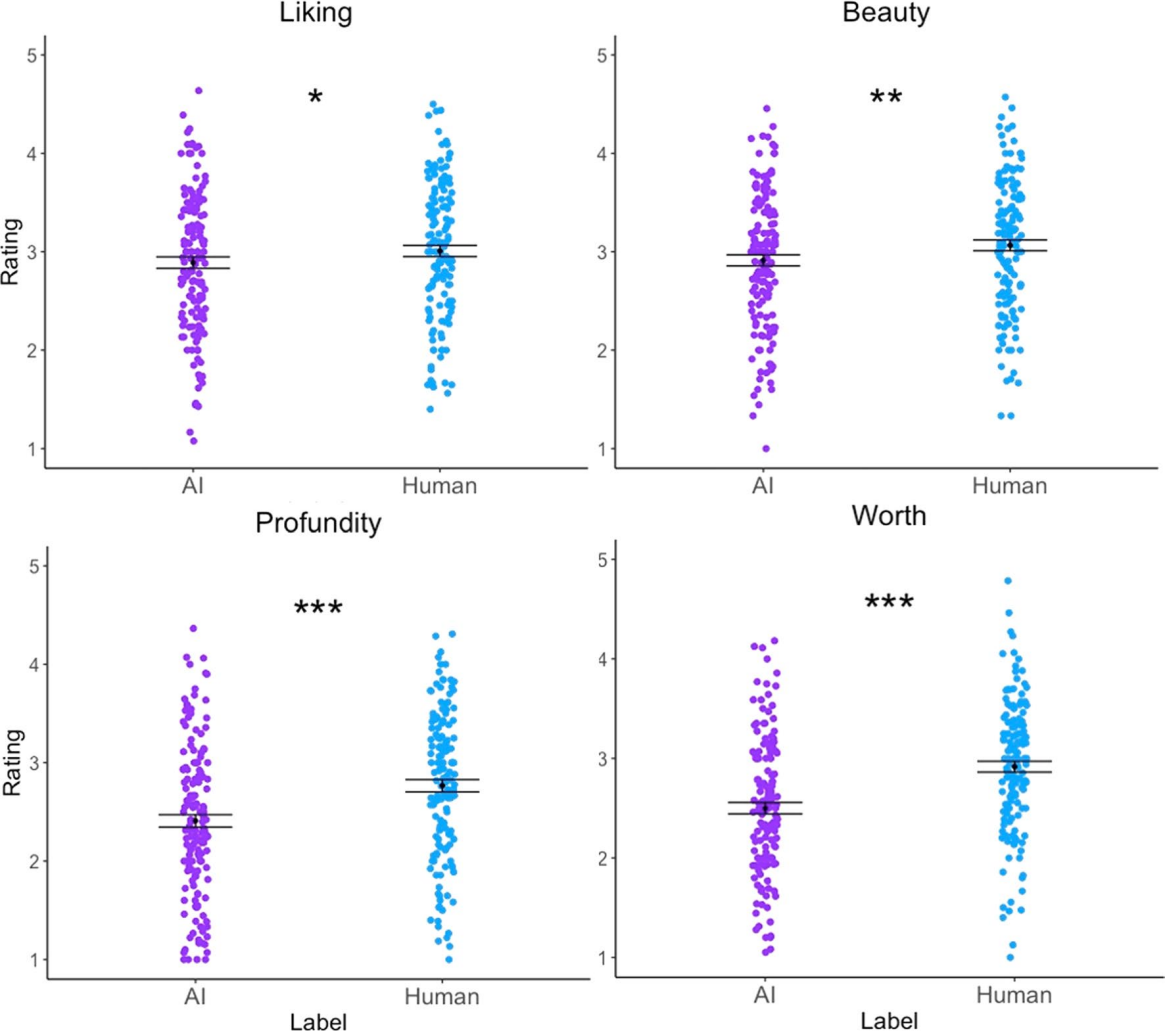
Distribution of values on artworks associated with AI and Human for each criterion (Liking, Beauty, Profundity, Worth). Error bars represent standard error of the mean. Following multiple-comparison corrections, *p < 0.05, **p < 0.01, ***p < 0.001

Paired *t*-tests with Bonferroni corrections show increased ratings for human-labelled over AI-labelled art for all four criteria: Liking (*t*(148) = 2.644, *p* = 0.036, *d* = 0.17), Beauty (*t*(148) = 3.499, *p* = 0.002, *d* = 0.22), Profundity (*t*(148) = 7.725, *p* < 0.001, *d* = 0.47), and Worth (*t*(148) = 10.042, *p* < 0.001, *d* = 0.61) (Fig. 2). In addition, for each criterion, a difference score between average human-labelled art rating and average AI-labelled art rating was calculated for each participant and was plotted against the participant’s CRT score. Only a relationship between CRT and Beauty difference scores emerged significant, with lower CRT scores associated with higher Beauty difference scores (that is, higher average Beauty judgements were given for human-labelled than AI-labelled art; *r* = − 0.17, *p* = 0.042).^2^ No such significant relationships emerged elsewhere (Liking: *p* = 0.07, Profundity: *p* = 0.95, Worth: *p* = 0.37). The same difference scores were also plotted against age; again, no significant relationships emerged (*p* = 0.29, *p* = 0.20, *p* = 0.26, *p* = 0.59). A significant relationship emerged between age and CRT performance (*r* = 0.23, *p* = 0.004), such that older participants performed better on the CRT.

Lastly, exploratory linear mixed-effects models using maximum likelihood estimation were performed for each of the four main criteria as the outcome variables: Liking, Beauty, Profundity, and Worth. Across models, participants and paintings were random effects, resulting in crossed random effects models. For all models, our manipulated variables of the art’s Label (AI or Human) and Painting Type (abstract or representational) were included, and we tested a potential interaction between Label and Painting Type. Across all criteria, a significant main effect of Painting Type emerged, such that participants preferred representational over abstract paintings regardless of Label (*p*’s < 0.001). For Liking, a non-significant main effect of Label (*p* = 0.057) was qualified by a significant interaction between Painting Type and Label (*p* = 0.041). Additionally, for Profundity, a main effect of Label (*p* < 0.001) was qualified by a significant interaction between Painting Type and Label (*p* = 0.036). When a painting is representational, art is liked more and found more profound when it is created by a human as opposed to AI. No significant interaction between Painting Type and Label emerged in Beauty (*p* = 0.21) or Worth (*p* = 0.18).

### Study 1 discussion

Across all four rating criteria (Liking, Beauty, Profundity, and Worth), we found an anti-AI bias, with participants showing a preference for art labelled as “human-created” compared to “AI-created.” This finding is particularly important given the within-subjects design of our study, its relatively large sample size, range of rating criteria, range of painting types, and randomization of labels across a large set of paintings.

Study 1 primarily served as a test of whether an anti-AI-art bias exists across a range of aesthetic judgement criteria. Given our relatively large sample, we were confident in the findings of this study, which, on the whole, mirrored findings of Chamberlain et al. (2018). However, extending these results, here we showed that this pattern of judgements for AI-art biases is reflected across appraisal processes outside of general aesthetic preferences. For instance, not only is AI-art liked less, but it is also viewed as less worthy and less profound, which may have interesting implications for the ways in which people will consume AI-art in the future.

In addition, though some studies also used a randomization of labels (as in the present study; e.g., Chiarella et al., 2022), our study was unique in its use of only AI-created images, across many more stimuli, in a within-subjects design. Statistically, this ensured higher power and increased confidence in our findings. In other words, participants were given the opportunity to judge both human- and AI-labelled artworks, and this permitted a sounder comparison of participants’ judgements across the two sources. Perhaps most importantly, though, was that all artworks were from AI in actuality. This underscores the fact that participants are generally incapable of noticing what source created what paintings—as supported by Gangadharbatla (2022) and Chamberlain et al. (2018)—which ultimately reflects the noteworthy quality of AI-art today. Though we did not explicitly ask participants for their confidence in the accuracy of our labels, our decision to exclusively select AI-created stimuli enabled us to dissociate top-down from bottom-up processing in these aesthetic judgements. That is, actual human versus actual AI images may differ in certain visual, bottom-up qualities.^3^ Thus, by exclusively selecting AI artwork and randomizing labels on a random subset of paintings, we ensured that any observed effects were necessarily top-down and isolated to source manipulation, as opposed to bottom-up, or driven by potential inherent differences across human- and AI-created artworks.

Somewhat surprising was the lack of statistically significant associations between our individual-differences measures (CRT and age) with the difference scores of human-AI ratings for each participant, which might be expected to track an AI bias. Although lower cognitive reflection scores were associated with higher Beauty preferences of human than AI art, no other significant relationship emerged between our individual-differences measures and the human-AI difference scores. Past work with the CRT has found negative relationships between CRT scores and perceived profundity of randomly generated statements (Pennycook et al., 2015), implying a relationship between cognitive reflection traits and subjective judgements of profundity that could extend to aesthetics. However, in Study 1, we found no support for this relationship. Given that the CRT assesses intuition-overriding in mathematical domains, we can conclude that (1) anti-AI biases largely do not rely on quantitative skills, (2) the anti-AI bias is not an intuitive response as probed by the mathematical problems in the CRT, and/or (3) we need more-sophisticated statistical analyses and/or power to uncover the true underlying relationships (if any) among these measures. In addition, the lack of statistically significant relationships between age and judgements of art was somewhat surprising. Though exploratory, this is at odds with some previous aesthetic studies that have reported age-dependent differences in judgements of art. Specifically, Mockros (1993) reported that general aesthetic judgements were rated higher by novice professional adults than novice undergraduates. More research is warranted on this topic given that we found no such age effect, which perhaps implies that younger participants—with their lives being more heavily dominated by AI than older participants—do not have different views on the creative products of AI than those of older adults.

## Study 2

The results from Study 1 provide initial insight into participant judgements of art that is believed to be generated either by AI algorithms or by humans. While not reliably predictable by individual differences such as age or CRT scores, a bias against AI art emerged. This bias, however, clearly depended on the criterion used to assess the artwork. Certainly, individuals do not just consider measures of Liking, Beauty, Profundity, and Worth when assessing artwork, but a host of other engagement processes.

This nuance to a general “anti-AI bias” matches with contemporary aesthetics models that argue that aesthetic judgements are rather complex. Indeed, largely pioneered by David Berlyne’s new experimental aesthetics (1960, 1971), art appreciation has been argued to be a consequence of elaborative appraisals of criteria like novelty, ambiguity, and complexity (which he deemed “collative” properties; Cupchik & Berlyn, 1979). Other emerging models have since provided more nuance to Berlyne’s traditional behaviorist approach (see Berlyne, 1975; Silvia, 2005), including more cognitive principles, and an increased understanding of the processes people utilize to engage with art. These models all agree in their proposal that humans engage in a multi-process evaluation of art, considering both sensory and non-sensory aspects (e.g., the Aesthetic Triad, Chatterjee & Vartanian, 2016; the pleasure-interest model, Graf & Landwehr, 2015; the information-processing model, Leder et al., 2004). Accordingly, many aesthetic properties—some more literal and surface-level, some non-sensory and more elaborative—can be probed during interactions with art. For instance, findings from Chamberlain et al. (2018) show that participants specifically cite that brush-strokes and other surface-level properties of the artistic process influence their evaluations of the final product. Moreover, when viewing anthropomorphized videos of robots painting, participants show greater appreciation for computer-generated art than when no anthropomorphized video of the robot is provided. This mirrors findings by Hong et al. (2022), who found that anthropomorphization of an AI-music algorithm led to higher acceptance of the algorithm as a true “musician,” which in turn led to increased aesthetic appreciation. Collectively, these studies suggest that there may be an implicit role of effortfulness or embodiment behind peoples’ evaluations of art. More specifically, it may be that peoples’ preferences for art increase as their beliefs about the amount of effort that went into creating a piece of art increase.

As informed by multi-level models as above, including additional judgement criteria could help us to shed further light on the reasons as to why people are making the aesthetic judgements they’re making. Thus, in addition to Liking, Beauty, Profundity, and Worth, in Study 2 we also included the following judgement criteria: emotionality (Emotion), perceived narrativity (Story), personal meaning (Meaningful), perceived effort (Effort), and estimated time to create (Time) as additional criteria for judgement. Importantly, while these now nine criteria may or may not behave in similar ways to one another, some may moderate ratings of others. In this way, we could gain insight on how levels of processing act interpedently or influence one another in this new sphere of aesthetic judgements with art created by non-humans. We specifically included these criteria given emotion’s prominent role in aesthetics and creativity (Silvia, 2005), the use of narratives as engines of consumption in other forms of art (a growing literature in music cognition investigates narratives in response to music, e.g., Margulis et al., 2019; McAuley et al., 2021), the act of deriving meaning from artwork that is often posited in models of aesthetics (e.g., Leder et al., 2004; Pelowski et al., 2017), and that effort and time involved in creation of a product is often a heuristic for quality (Kruger et al., 2004).

In addition, we extended the battery of individual-differences measures given our mostly null results from the individual-differences measures in Study 1. While we choose to keep both age and CRT scores as potential individual differences, we also included empathy skills (to determine if participants differ in judgement based on the ability to empathize with other agents, including AI, perhaps explaining AI-art judgements), openness to experience (given its role in aesthetic encounters; Kaufman, 2013; McCrae, 2007; McCrae & Greenberg, 2014; Silvia et al., 2015), personal attitudes toward AI (which we hypothesized could predict judgements of artwork made by AI), and facets of the creative mindset scale (CMS; Karwowski, 2014), which include growth and fixed mindsets. The CMS specifically asks about views of who can produce products of creativity, and we thus deemed it important to include in this study to determine if growth mindsets lead to higher AI-art ratings as we hypothesized. In addition, to detect more sensitive relationships between both individual-differences measures and judgement criteria, we aimed to use more-sophisticated statistical modeling with a pre-registered design. In sum, through implementing linear mixed models, we sought to determine whether we would replicate and explain the anti-AI effect with wider criteria of judgements—to parallel common multi-level processing models of aesthetics—and an extension of individual-differences measures.

### Method

Study 2’s design and analysis plan were pre-registered. Study 2 was approved by the Duke University Campus Institutional Review Board (2022-0534). All data and stimuli, and the pre-registration, can be found at https://osf.io/cgw8v/.

#### Participants

One-hundred and fifty-one participants were recruited through Prolific.^4^ Only native English speakers with at least a 90% approval rate were permitted to enroll in the study. Like Study 1, they were told at sign-up that they would be evaluating pieces of art and filling out questionnaires meant to probe the way they think. Participants were debriefed following completion of the study and were compensated for their time. As per our pre-registration, data from two participants were excluded for failing an attention check (age and birth-year needed to correspond appropriately), and one participant was excluded for bad data, resulting in 148 participants (*M*_age_ = 36.66, *SD* = 12.73; female = 74, non-binary = 2).

#### Materials

The same 30 images from Study 1 were presented in random order in Study 2, again with a randomly assigned label of “human-created” or “AI-created.” Ratings were done on the same criteria: Liking, Beauty, Profundity, Worth; note that the phrasing for the Profundity question in this study changed from “How profound or meaningful is this image?” to “How profound is this artwork?” for simplicity. Participants rated the images on five additional criteria as well: “To what extent does this artwork elicit an emotional response in you?” (Emotion), “To what extent can you imagine a story communicated through this artwork?” (Story), “To what extent do you find this artwork personally meaningful?” (Meaningful), “How much effort do you believe was involved in making this artwork?” (Effort), and “How much time do you believe it took to create this piece?” (Time). All questions were on a 1–5 Likert [“None/not at all”… “Very much”] except Time, which was a free-response question that allowed for numeric entry in a “Days,” “Hours,” and “Minutes” field. Participants were told to simply enter “0” if a certain field did not apply. Lastly, for each image, participants were asked the Label-Check question from Study 1 for trial-based exclusions: “Based on the label above this image, was this image created by a human or an artificial intelligence computer program?” Those who did not provide the correct answer had that trial eliminated, resulting in 114 trials (of 4440 total trials across the 148 participants) excluded from analyses. As in Study 1, no participants were removed on the basis of this attention check, only trials. This specific exclusion criterion was not specified in our original pre-registration plan, though, for purposes of consistency, it follows the procedure from Study 1.

Before the ratings, participants filled out various questionnaires. Participants completed the same CRT as in Study 1. In addition, to probe trait-level empathy skills, participants completed the Toronto Empathy Questionnaire (TEQ; Spreng et al., 2009). The TEQ has been demonstrated to be a highly internally consistent (*⍺* = 0.87) and reliable (*r* = 0.81) self-report empathy measure. The 16-item questionnaire was completed on a 1–5 Likert [“Never”… “Always”], and responses were reverse-scored when appropriate and summed for a final empathy score.

Participants also completed the General Attitudes toward Artificial Intelligence Scale (GAAIS) to assess positive and negative sentiments of AI in general (Schepman & Rodway, 2020). The 20-item GAAIS has been fully validated and found to report a consistent positive subscale (*⍺* = 0.88) and negative subscale (*⍺* = 0.82), with strong convergent validity with other AI-related measures (Schepman & Rodway, 2022). Scores for each subscore were aggregated through summation, producing a “Positive Attitude” score and a “Negative Attitude” score.

To probe participant beliefs about creativity, we administered the Creative Mindset Scale (CMS: Karwowski, 2014). The CMS assesses how much an individual believes creativity can be developed and trained through time (i.e., “growth” mindset) and how much they believe creativity is static and innate (i.e., “fixed” mindset). Participants rated the 10 items of the CMS on a 1–5 Likert Scale [“Definitely not”… “Definitely yes”]. Scores for each mindset were aggregated through summation, producing a “Growth” value and a “Fixed” value.

Participants also completed the 12-item “Openness to Experience” subset of the NEO-Five Factor Personality Inventory. Though the full NEO-FFI can also assess Consciousness, Extraversion, Agreeableness, and Neuroticism, we chose to implement only the Openness subset given its previous specific involvement in aesthetic encounters (Kaufman, 2013; McCrae, 2007; McCrae & Greenberg, 2014; Silvia et al., 2015), and to reduce study length. Items were answered on a 1–5 Likert scale [“Strongly disagree”… “Strongly agree”] and were reverse-scored when appropriate and aggregated through summing.

Lastly, in an effort to guide future research, we had participants answer a series of exploratory, free-response and single-item questions. These data are listed in our pre-registration as “exploratory,” and, for the sake of clarity and brevity, they are not reported here.

#### Procedure

Upon direction to the Qualtrics survey, participants gave informed consent on the nature of the study. Then, they were given two bot checks; no participant failed, and all moved forward with the study. Participants were then directed to complete demographic information, followed by the CRT, TEQ, openness from the NEO-FFI, CMS, GAAIS, and additional single-item questions, in that order. Importantly, participants were specifically asked their age in the demographic questionnaire, and at the end of the study, were asked their year of birth; this served as an attention check. As per our pre-registration, data from two participants with incongruent birth years and ages were excluded from our analyses. Following questionnaires, participants completed the ratings of the images, after which they were debriefed and redirected to Prolific.

### Statistical analyses

Linear mixed-effects models using maximum likelihood estimation were estimated in *R* (version 4.2.0; *R* Core Team, 2022) using the {lme4} package (version 1.1.33; Bates et al., 2015). A mixed effect model was estimated for each of the four main criteria from Study 1 as the outcome variables: Liking, Beauty, Profundity, and Worth. The linear mixed-effects models diverged from our pre-registration with the use of frequentist rather than Bayesian models.^5^ Power analyses were not performed a priori; instead, the sample size was determined using the same procedure reported in our Study 1.

For all models, our manipulated variables of the art’s Label (AI or Human) and Painting Type (abstract or representational) were included. Both Label and Painting Type were fixed-effect variables. We did not include the interaction between Label and Painting Type in our reports because this term was not significant in any of the models. Across models, participants and paintings were random effects, resulting in crossed random effects models. For participants, we included the random intercept and Label slope to model people’s differences in outcome related to Label. For paintings, we only included the random intercept term to account for differences in the level of the outcome for each painting.

For Model 1, we focused on individual-differences measures to identify characteristics of who were related to our outcome variables. All individual-differences measures were continuous (i.e., sum scores) fixed effect variables. Each individual-difference measure was mean-centered to increase the interpretability of the model (e.g., each intercept represented the mean of each outcome). Interactions between Label and all individual-differences measures were specified in this model.

For Model 2, we added the five additional criteria (Emotion, Story, Meaningful, Effort, and Time), as well as their respective interactions with Label, as predictors to the model. This model represented aspects of why outcomes might be rated differently. Time was log-transformed due to its logarithmic distribution. All additional criteria were level-1 or within-painting variables. Since Model 2 represents the full model, the equation is presented here:

$$\begin{aligned} {\varvec{L}}1:{Y}_{ij}&= {\beta }_{0j}+ {\beta }_{1j}PaintingTyp{e}_{ij}+ {\beta }_{2j}Labe{l}_{ij}+ {\beta }_{3j}Opennes{s}_{ij}+ {\beta }_{4j}PositiveA{I}_{ij}+ {\beta }_{5j}NegativeA{I}_{ij}\\&\quad+{\beta }_{6j}GrowthMindse{t}_{ij}+ {\beta }_{7j}FixedMindse{t}_{ij}+ {\beta }_{8j}CorrectCR{T}_{ij}+ {\beta }_{9j}Ag{e}_{ij}\\&\quad+ {\beta }_{10j}Emotio{n}_{ij}+ {\beta }_{11j}Stor{y}_{ij}+ {\beta }_{12j}Meaningfu{l}_{ij}+ {\beta }_{13j}Effor{t}_{ij}+ {\beta }_{14j}\mathrm{log}Tim{e}_{ij}\\&\quad+{\beta }_{15j}Labe{l}_{ij}\times Opennes{s}_{ij}+{\beta }_{16j}Labe{l}_{ij}\times PositiveA{I}_{ij}+{\beta }_{17j}Labe{l}_{ij}\times NegativeA{I}_{ij}+{\beta }_{18j}Labe{l}_{ij}\times GrowthMindse{t}_{ij}+{\beta }_{19j}Labe{l}_{ij}\times FixedMindse{t}_{ij}\\&\quad+{\beta }_{20j}Labe{l}_{ij}\times CorrectCR{T}_{ij}+ {\beta }_{21j}Labe{l}_{ij}\times Ag{e}_{ij}+ {\beta }_{22j}Labe{l}_{ij}\times Emotio{n}_{ij}+{\beta }_{23j}Labe{l}_{ij}\times Stor{y}_{ij}\\&\quad+ {\beta }_{24j}Labe{l}_{ij}\times Meaningfu{l}_{ij}+ {\beta }_{25j}Labe{l}_{ij}\times Effor{t}_{ij}+ {\beta }_{26j}Labe{l}_{ij}\times \mathrm{log}Tim{e}_{ij}\\&\quad+{Participan{t}_{oj}}_{i}+{Painting}_{1j}+{\epsilon }_{ij}\end{aligned}$$

$${\varvec{L}}2: Participan{t}_{0j}= {\gamma }_{00}+ {\gamma }_{01}Labe{l}_{oj}+ {u}_{0j}$$

$${\varvec{L}}2:Paintin{g}_{1j}= {\gamma }_{10}+{u}_{1j}$$

For Model 3, we included only the significant predictors from Model 2. For each model, we report model fit using Akaike’s information criterion (AIC) and variance explained using marginal ${R}^{2}$ (fixed effects only), intraclass correlation (ICC; random effects only), and conditional ${R}^{2}$ (all effects combined). For brevity, we focus our report on Model 3 but provide all models’ results in Tables 1–4 to be comprehensive.

### Study 2 results

#### Liking

**Fig. 3:**
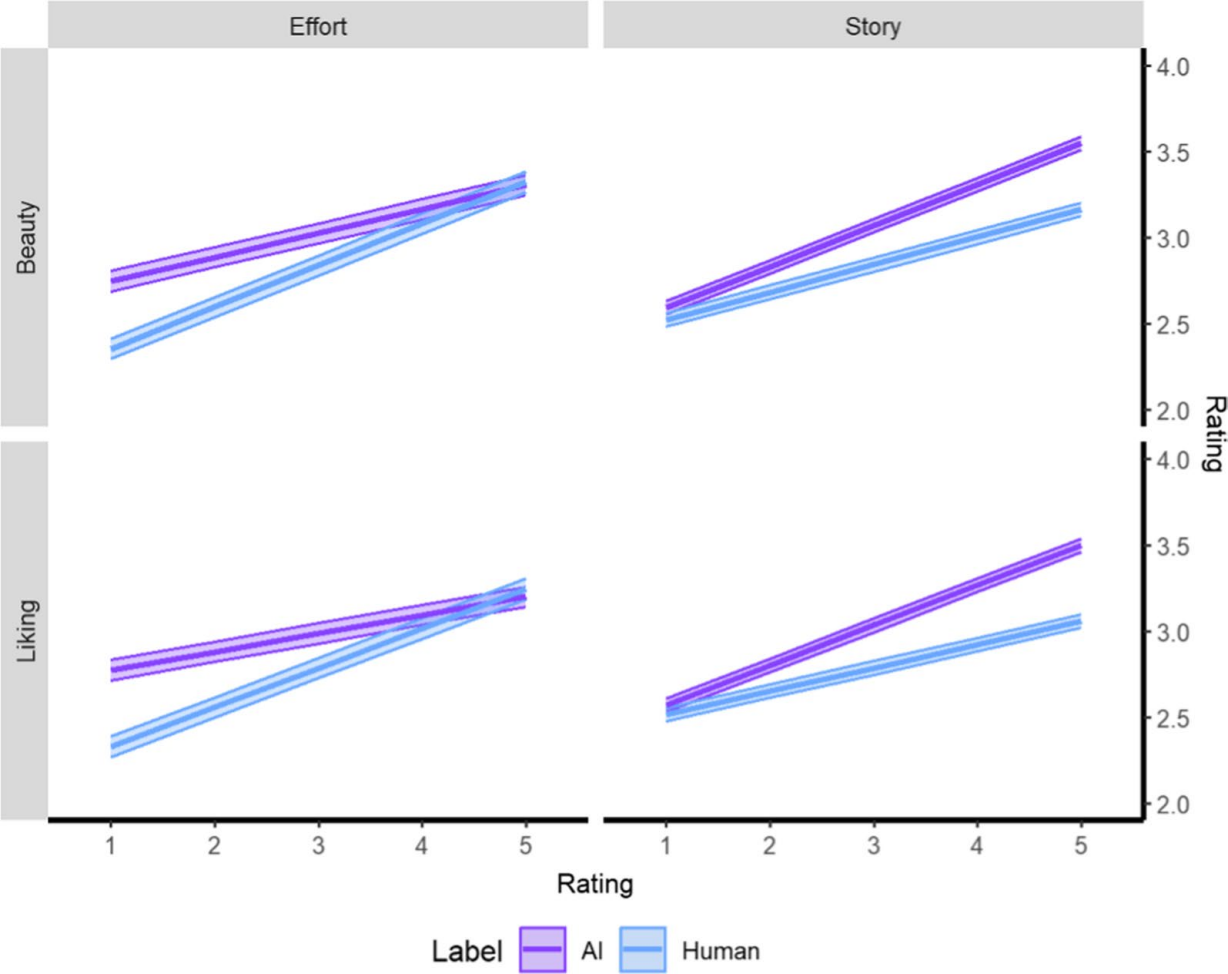
Interaction plots showing moderations by Effort and Story on Liking and Beauty, two sensory-level criteria. Confidence bands represent the 95% confidence interval

Results for the Liking models are reported in Table 1. There were two interactions involving Label: Story (*b* = − 0.09, *p* < 0.001) and Effort (*b* = 0.12, *p* < 0.001). For Story, Liking was greater when Label was “AI” than when Label was “human” and this difference increased as Story increased (Fig. 3). Based on Fig. 3 and the difference between the conditional means, for low-to-moderate values of Effort (ratings = 1–4; Fig. 3), Liking was greater when Label was “AI” than when Label was “human.” For high values of Effort (rating = 5), Liking was greater when Label was “human” than when Label was “AI.” There were main effects for Emotion (*b* = 0.32, *p* < 0.001), Meaningful (*b* = 0.24, *p* < 0.001), and Painting Type (*b* = 0.28, *p* < 0.001). For Emotion and Meaningful, Liking increased as these criteria increased. For Painting Type, Liking was greater for representational paintings than abstract paintings. The fixed effects of the model explained 57.9% of the variance, the random effects explained 32.0% of the variance, and all effects explained 71.2% of the variance.

**Table 1:** Estimates and associated CIs predicting “Liking” judgements

Liking	Model 1	Model 2	Model 3
*Fixed effects* *[95% CI]*			
(Intercept)	***2.38*** ***[2.17, 2.60]***	***0.55*** ***[0.38, 0.71]***	***0.57*** ***[0.41, 0.73]***
Label (Human = 1)	***0.24*** ***[0.15, 0.32]***	*− 0.26* *[− 0.45, − 0.07]*	***− 0.28*** ***[− 0.43, − 0.13]***
Painting Type (Representational = 1)	***0.75*** ***[0.48, 1.01]***	***0.28*** ***[0.13, 0.44]***	***0.28*** ***[0.13, 0.44]***
Openness	0.01 [− 0.01, 0.03]	0.01 [− 0.00, 0.03]	–
Positive AI	*0.03* *[0.01, 0.04]*	0.00 [− 0.01, 0.01]	–
Negative AI	0.01 [− 0.01, 0.03]	0.01 [− 0.00, 0.02]	–
Growth	*0.06* *[0.01, 0.11]*	0.01 [− 0.03, 0.04]	–
Fixed	*0.06* *[0.02, 0.09]*	0.02 [− 0.01, 0.04]	–
Empathy	0.00 [− 0.01, 0.02]	− 0.00 [− 0.01, 0.01]	–
CRT	− 0.04 [− 0.09, 0.01]	0.01 [− 0.03, 0.04]	–
Age	− 0.00 [− 0.01, 0.01]	− 0.01 [− 0.01, 0.00]	–
Emotion	–	***0.34*** ***[0.29, 0.39]***	***0.32*** ***[0.29, 0.36]***
Story	–	***0.23*** ***[0.19, 0.27]***	***0.23*** ***[0.19, 0.27]***
Meaningful	–	***0.20*** ***[0.14, 0.25]***	***0.24*** ***[0.20, 0.27]***
Effort	–	***0.11*** ***[0.06, 0.15]***	***0.11*** ***[0.07, 0.15]***
Time (log)	–	0.01 [− 0.00, 0.03]	–
Label × Openness	0.00 [− 0.01, 0.02]	− 0.01 [− 0.02, 0.00]	–
Label × Positive AI	*− 0.02* *[− 0.03, − 0.00]*	− 0.01 [− 0.01, 0.00]	–
Label × Negative AI	− 0.01 [− 0.02, 0.01]	0.00 [− 0.01, 0.01]	–
Label × Growth	0.03 [− 0.01, 0.07]	0.01 [− 0.02, 0.04]	–
Label × Fixed	0.01 [− 0.02, 0.04]	0.01 [− 0.01, 0.03]	–
Label × Empathy	0.00 [− 0.01, 0.01]	0.00 [− 0.01, 0.01]	–
Label × CRT	− 0.03 [− 0.06, 0.01]	0.00 [− 0.03, 0.03]	–
Label × Age	− 0.00 [− 0.01, 0.01]	− 0.00 [− 0.01, 0.00]	–
Label × Emotion	–	− 0.04 [− 0.10, 0.03]	–
Label × Story	–	***− 0.10*** ***[− 0.15, − 0.04]***	***− 0.09*** ***[− 0.14, − 0.05]***
Label × Meaningful	–	0.06 [− 0.00, 0.13]	–
Label × Effort	–	***0.12*** ***[0.06, 0.18]***	***0.12*** ***[0.07, 0.17]***
Label × Time (log)	–	− 0.01 [− 0.04, 0.01]	–
*Random effects*			
Participant (Intercept)	0.44	0.19	0.21
Label (Slope)	0.15	0.05	0.06
Painting (Intercept)	0.13	0.04	0.04
Residual	0.95	0.53	0.53
*Model*			
Marginal	0.16	0.59	0.58
ICC	0.37	0.30	0.32
Conditional	0.47	0.72	0.71
AIC	12,819.13	10,300.48	10,126.86

*Note*. CI = confidence interval, Marginal = variance explained by fixed effects, ICC = intraclass correlation or variance explained by random effects, Conditional = variance explained by fixed and random effects, AIC = Akaike Information Criterion. Italicized text = *p* < 0.05, bolded text = *p* < 0.001

#### Beauty

Results for the Beauty models are reported in Table 2. Similar to Liking, there were interactions involving Label: Story (*b* = − 0.08, *p* < 0.001) and Effort (*b* = 0.10, *p* < 0.001). The effects similarly parallel Liking in that Beauty was greater when Label was “AI” than when Label was “human” and this difference increased as Story increased and for low-to-moderate values of Effort (ratings = 1–4; Fig. 3), Beauty was greater when Label was “AI” than when Label was “human” whereas the reverse was found for high values of Effort (rating = 5, Fig. 3). There was an additional interaction between Label and Openness to Experience (*b* = − 0.01, *p* = 0.003) such that well below average Openness to Experience (− 20) had higher Beauty for the human Label than the AI Label but this difference reversed with below average (− 10) Openness to Experience and higher such that Beauty was higher for the AI Label than the human Label. Similar to Liking, there were main effects for Emotion (*b* = 0.23, *p* < 0.001), Meaningful (*b* = 0.23, *p* < 0.001), and Painting Type (*b* = 0.32, *p* < 0.001) such that Beauty increased as Emotion and Meaningful increased and Beauty was greater for representational paintings than abstract paintings. There was an additional main effect of Age (*b* = − 0.01, *p* = 0.007) such that people younger than average (-20) rated Beauty higher than people older than average (40). The fixed effects of the model explained 53.1% of the variance, the random effects explained 34.0% of the variance, and all effects explained 69.0% of the variance.

**Table 2:** Estimates and associated CIs predicting “Beauty” judgements

Beauty	Model 1	Model 2	Model 3
*Fixed effects* *[95% CI]*			
(Intercept)	***2.43*** ***[2.20, 2.67]***	***0.75*** ***[0.56, 0.95]***	***0.74*** ***[0.55, 0.93]***
Label (Human = 1)	***0.23*** ***[0.15, 0.31]***	***− 0.37*** ***[− 0.57, − 0.18]***	***− 0.28*** ***[− 0.43, − 0.13]***
Painting Type (Representational = 1)	***0.75*** ***[0.46, 1.05]***	*0.32* *[0.11, 0.52]*	*0.32* *[0.11, 0.52]*
Openness	0.01 [− 0.01, 0.03]	*0.02* *[0.00, 0.03]*	0.01 [− 0.00, 0.02]
Positive AI	*0.02* *[0.01, 0.03]*	0.00 [− 0.01, 0.01]	–
Negative AI	0.01 [− 0.01, 0.03]	0.01 [− 0.00, 0.03]	–
Growth	0.04 [− 0.01, 0.09]	− 0.01 [− 0.05, 0.02]	–
Fixed	*0.05* *[0.01, 0.08]*	0.01 [− 0.02, 0.04]	–
Empathy	− 0.00 [− 0.02, 0.01]	− 0.01 [− 0.02, 0.00]	–
CRT	− 0.04 [− 0.06, 0.04]	0.03 [− 0.00, 0.07]	–
Age	− 0.01 [− 0.02, 0.01]	*− 0.01* *[− 0.02, − 0.00]*	*− 0.01* *[− 0.01, − 0.00]*
Emotion	–	***0.24*** ***[0.19, 0.29]***	***0.23*** ***[0.19, 0.26]***
Story	–	***0.24*** ***[0.20, 0.28]***	***0.24*** ***[0.20, 0.28]***
Meaningful	–	***0.21*** ***[0.15, 0.26]***	***0.23*** ***[0.20, 0.27]***
Effort	–	***0.14*** ***[0.10, 0.19]***	***0.14*** ***[0.10, 0.18]***
Time (log)	–	− 0.00 [− 0.02, 0.02]	–
Label × Openness	− 0.01 [− 0.02, 0.01]	*− 0.01* *[− 0.02, 0.00]*	*− 0.01* *[− 0.02, − 0.00]*
Label × Positive AI	*− 0.01* *[− 0.03, − 0.00]*	− 0.00 [− 0.01, 0.00]	–
Label × Negative AI	− 0.01 [− 0.02, 0.01]	− 0.00 [− 0.01, 0.01]	–
Label × Growth	0.02 [− 0.02, 0.05]	0.00 [− 0.03, 0.03]	–
Label × Fixed	− 0.01 [− 0.03, 0.02]	− 0.01 [− 0.03, 0.01]	–
Label × Empathy	0.00 [− 0.01, 0.01]	− 0.00 [− 0.01, 0.01]	–
Label × CRT	− 0.03 [− 0.06, 0.01]	0.00 [− 0.03, 0.03]	–
Label × Age	− 0.00 [− 0.01, 0.01]	0.00 [− 0.00, 0.01]	–
Label × Emotion	–	− 0.02 [− 0.09, 0.04]	–
Label × Story	–	*− 0.19* *[− 0.14, − 0.03]*	***− 0.08*** ***[− 0.12, − 0.04]***
Label × Meaningful	–	0.04 [− 0.02, 0.11]	–
Label × Effort	–	*0.09* *[0.03, 0.15]*	***0.10*** ***[0.05, 0.16]***
Label × Time (log)	–	0.01 [− 0.01, 0.04]	–
*Random effects*			
Participant (Intercept)	0.44	0.21	0.24
Label (Slope)	0.13	0.05	0.05
Painting (Intercept)	0.16	0.08	0.08
Residual	0.90	0.55	0.55
*Model*			
Marginal	0.13	0.53	0.53
ICC	0.40	0.33	0.34
Conditional	0.48	0.69	0.69
AIC	12,606.95	10,449.15	10,300.65

*Note*. CI = confidence interval, Marginal = variance explained by fixed effects, ICC = intraclass correlation or variance explained by random effects, Conditional = variance explained by fixed and random effects, AIC = Akaike Information Criterion. Italicized text = *p* < 0.05, bolded text = *p* < 0.001

#### Profundity

**Fig. 4:**
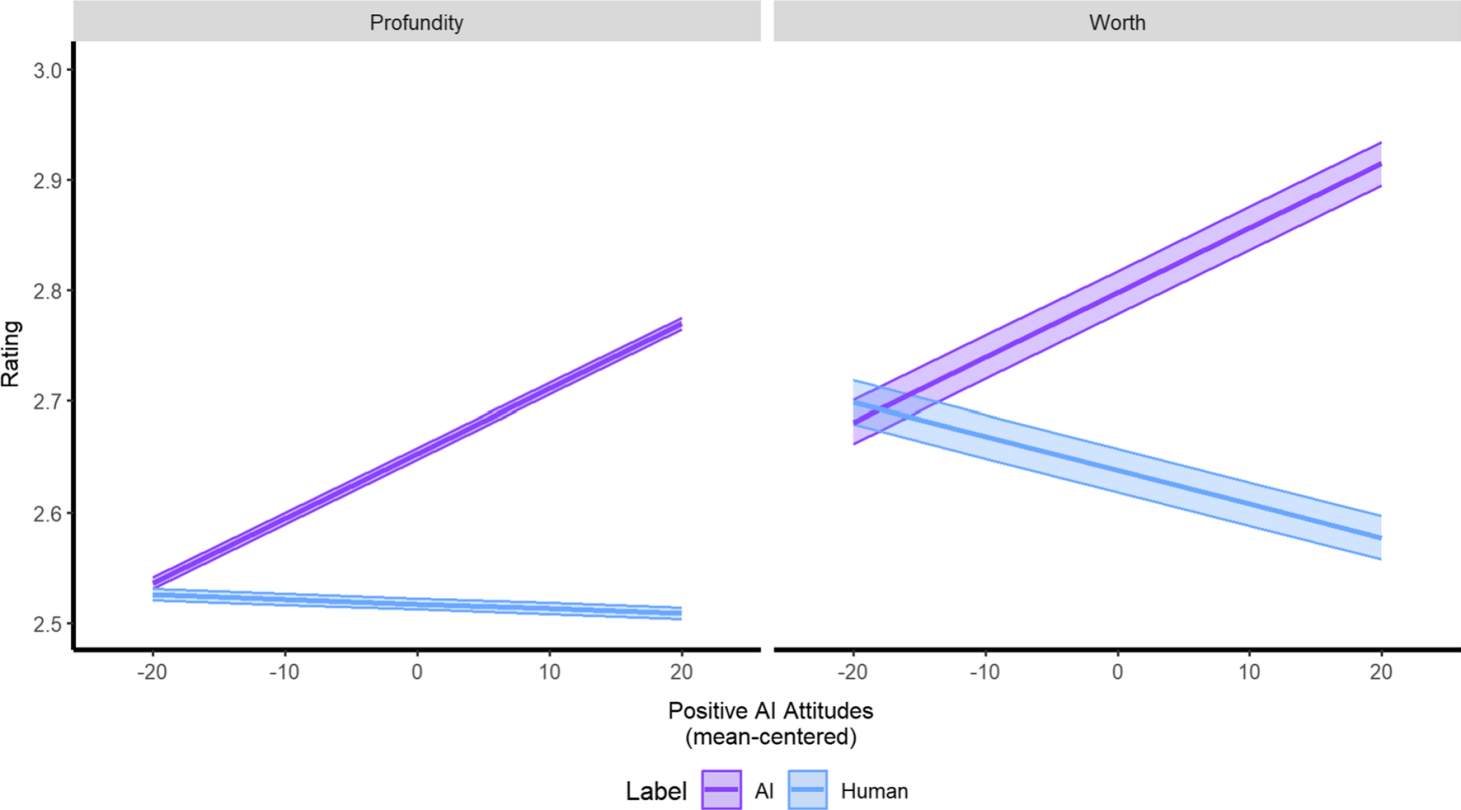
Interaction plot of Positive AI attitudes on communicative judgements of Profundity and Worth. Confidence bands represent the 95% confidence interval

Results for the Profundity models are reported in Table 3. There was one interaction involving Label and Positive AI attitudes (*b* = − 0.01, *p* = 0.016) such that, based on Fig. 4 and the difference between the conditional means, there was slightly higher Profundity when Label was “AI” and Positive AI attitudes were well below average (− 20; Fig. 4). This difference grew larger as Positive AI attitudes increased to well above average (20). There were main effects for all five additional criteria (Emotion: *b* = 0.22, *p* < 0.001; Story: *b* = 0.19, *p* < 0.001; Meaningful: *b* = 0.26, *p* < 0.001; Effort: *b* = 0.19, *p* < 0.001; Time (log): *b* = 0.02, *p* = 0.011) such that Profundity increased as each criterion increased. For both growth (*b* = 0.04, *p* = 0.005) and fixed (*b* = 0.02, *p* = 0.017) mindsets, Profundity increased as these characteristics went from well below average (− 10 and − 8, respectively) to well above average (6 and 10, respectively). The fixed effects of the model explained 63.5% of the variance, the random effects explained 26.0% of the variance, and all effects explained 73.0% of the variance.

**Table 3:** Estimates and associated CIs predicting “Profundity” judgements

Profundity	Model 1	Model 2	Model 3
*Fixed effects* *[95% CI]*			
(Intercept)	***2.20*** ***[2.02, 2.38]***	***0.42*** ***[0.31, 0.53]***	***0.40*** ***[0.31 0.50]***
Label (Human = 1)	***0.37*** ***[0.29, 0.58]***	− 0.15 [− 0.31, 0.01]	***− 0.14*** ***[− 0.20, − 0.07]***
Painting Type (Representational = 1)	***0.40*** ***[0.21, 0.58]***	− 0.04 [− 0.11, 0.02]	–
Openness	− 0.01 [− 0.03, 0.01]	− 0.00 [− 0.01, 0.01]	–
Positive AI	*0.02* *[0.01, 0.04]*	0.01 [− 0.00, 0.01]	0.01 [− 0.00, 0.01]
Negative AI	0.00 [− 0.02, 0.02]	0.00 [− 0.01, 0.01]	–
Growth	***0.11*** ***[0.05, 0.16]***	*0.04* *[0.02, 0.07]*	*0.04* *[0.01, 0.06]*
Fixed	*0.07* *[0.03, 0.11]*	*0.02* *[0.00, 0.04]*	*0.02* *[0.00, 0.04]*
Empathy	0.00 [− 0.01, 0.02]	− 0.00 [− 0.01, 0.01]	–
CRT	*− 0.08* *[− 0.13, − 0.02]*	− 0.03 [− 0.06, 0.00]	–
Age	0.01 [− 0.00, 0.02]	0.00 [− 0.00, 0.01]	–
Emotion	–	***0.22*** ***[0.18, 0.26]***	***0.22*** ***[0.19, 0.25]***
Story	–	***0.18*** ***[0.15, 0.21]***	***0.19*** ***[0.17, 0.22]***
Meaningful	–	***0.26*** ***[0.22, 0.31]***	***0.26*** ***[0.22, 0.29]***
Effort	–	***0.20*** ***[0.16, 0.24]***	***0.19*** ***[0.16, 0.22]***
Time (log)	–	*0.02* *[0.00, 0.03]*	*0.02* *[0.00, 0.03]*
Label × Openness	0.01 [− 0.01, 0.02]	− 0.01 [− 0.01, 0.00]	–
Label × Positive AI	*− 0.01* *[− 0.03, − 0.01]*	*− 0.01* *[− 0.01, − 0.00]*	*− 0.01* *[− 0.01, − 0.00]*
Label × Negative AI	− 0.01 [− 0.02, 0.01]	0.00 [− 0.00, 0.01]	–
Label × Growth	− 0.00 [− 0.04, 0.03]	− 0.01 [− 0.03, 0.01]	–
Label × Fixed	− 0.01 [− 0.03, 0.02]	− 0.00 [− 0.02, 0.01]	–
Label × Empathy	0.01 [− 0.00, 0.02]	0.00 [− 0.00, 0.01]	–
Label × CRT	− 0.02 [− 0.06, 0.01]	0.00 [− 0.02, 0.02]	–
Label × Age	− 0.00 [− 0.01, 0.00]	− 0.00 [− 0.01, 0.00]	–
Label × Emotion	–	− 0.00 [− 0.06, 0.06]	–
Label × Story	–	0.03 [− 0.01, 0.08]	–
Label × Meaningful	–	− 0.01 [− 0.07, 0.05]	–
Label × Effort	–	− 0.02 [− 0.07, 0.04]	–
Label × Time (log)	–	− 0.00 [− 0.02, 0.02]	–
*Random effects*			
Participant (Intercept)	0.53	0.13	0.13
Label (Slope)	0.12	0.02	0.02
Painting (Intercept)	0.06	0.00	0.01
Residual	0.76	0.41	0.41
*Model*			
Marginal	0.15	0.64	0.64
ICC	0.44	0.25	0.26
Conditional	0.52	0.73	0.73
AIC	11,905.68	9098.20	8940.49

*Note*. CI = confidence interval, Marginal = variance explained by fixed effects, ICC = intraclass correlation or variance explained by random effects, Conditional = variance explained by fixed and random effects, AIC = Akaike Information Criterion. Italicized text = *p* < 0.05, bolded text = *p* < 0.001

#### Worth

Results for the Worth models are reported in Table 4. Similar to Profundity, there was one interaction involving Label and Positive AI attitudes (*b* = -0.01, *p* = 0.014) but it differed such that there was slightly higher Profundity when Label was “human” and Positive AI attitudes were well below average (− 20; Fig. 4). This difference reversed as Positive AI attitudes went from well below average (− 20) to below average (− 10) and continued to diverge to well above average (20). Also similar to Profundity, there were main effects for all five additional criteria (Emotion: *b* = 0.18; Story: *b* = 0.12; Meaningful: *b* = 0.15; Effort: *b* = 0.27; Time (log): *b* = 0.05; all *p*’s < 0.001) such that Profundity increased as each criterion increased. The fixed effects of the model explained 57.4% of the variance, the random effects explained 32.0% of the variance, and all effects explained 71.3% of the variance.

**Table 4.** Estimates and associated CIs predicting “Worth” judgements

Worth	Model 1	Model 2	Model 3
*Fixed effects* *[95% CI]*			
(Intercept)	***2.29*** ***[2.12, 2.45]***	***0.65*** ***[0.53, 0.77]***	***0.66*** ***[0.55, 0.77]***
Label (Human = 1)	***0.52*** ***[0.43, 0.61]***	*− 0.19* *[− 0.37, − 0.02]*	*− 0.21* *[− 0.35, − 0.07]*
Painting Type (Representational = 1)	***0.35*** ***[0.18, 0.52]***	− 0.01 [− 0.09, 0.06]	–
Openness	− 0.01 [− 0.03, 0.01]	0.01 [− 0.01, 0.02]	–
Positive AI	*0.02* *[0.00, 0.03]*	0.00 [− 0.01, 0.01]	0.01 [− 0.00, 0.01]
Negative AI	0.01 [− 0.01, 0.03]	0.01 [− 0.00, 0.02]	–
Growth	*0.07* *[0.02, 0.12]*	0.01 [− 0.02, 0.04]	–
Fixed	*0.05* *[0.01, 0.09]*	0.01 [− 0.01, 0.03]	–
Empathy	0.00 [− 0.01, 0.02]	− 0.00 [− 0.01, 0.01]	–
CRT	*− 0.08* *[− 0.13, − 0.03]*	− 0.03 [− 0.06, 0.00]	–
Age	0.00 [− 0.01, 0.01]	− 0.00 [− 0.01, 0.01]	–
Emotion	–	***0.17*** ***[0.13, 0.21]***	***0.18*** ***[0.15, 0.20]***
Story	–	***0.14*** ***[0.11, 0.17]***	***0.12*** ***[0.10, 0.14]***
Meaningful	–	***0.18*** ***[0.14 0.23]***	***0.15*** ***[0.12, 0.18]***
Effort	–	***0.25*** ***[0.21, 0.29]***	***0.27*** ***[0.23, 0.31]***
Time (log)	–	***0.04*** ***[0.02, 0.06]***	***0.05*** ***[0.03, 0.06]***
Label × Openness	0.01 [− 0.01, 0.03]	− 0.00 [− 0.01, 0.01]	–
Label × Positive AI	*− 0.02* *[− 0.03, − 0.00]*	*− 0.01* *[− 0.02, − 0.00]*	*− 0.01* *[− 0.02, − 0.00]*
Label × Negative AI	− 0.01 [− 0.03, 0.01]	− 0.00 [− 0.01, 0.01]	–
Label × Growth	0.00 [− 0.04, 0.05]	0.00 [− 0.03, 0.02]	–
Label × Fixed	− 0.01 [− 0.04, 0.02]	0.00 [− 0.02, 0.02]	–
Label × Empathy	0.00 [− 0.01, 0.02]	0.00 [− 0.01, 0.01]	–
Label × CRT	− 0.02 [− 0.06, 0.03]	− 0.00 [− 0.03, 0.02]	–
Label × Age	− 0.01 [− 0.01, 0.00]	− 0.00 [− 0.01, 0.00]	–
Label × Emotion	–	0.01 [− 0.04, 0.07]	–
Label × Story	–	− 0.04 [− 0.09, 0.00]	–
Label × Meaningful	–	− 0.06 [− 0.11, 0.00]	–
Label × Effort	–	*0.06* *[0.00, 0.11]*	0.02 [− 0.02, 0.06]
Label × Time (log)	–	0.01 [− 0.01, 0.04]	–
*Random effects*			
Participant (Intercept)	0.47	0.16	0.17
Label (Slope)	0.24	0.10	0.10
Painting (Intercept)	0.05	0.01	0.01
Residual	0.61	0.36	0.36
*Model*			
Marginal	0.16	0.59	0.57
ICC	0.45	0.31	0.32
Conditional	0.54	0.72	0.71
AIC	11,040.92	8711.31	8557.09

*Note*. CI = confidence interval, Marginal = variance explained by fixed effects, ICC = intraclass correlation or variance explained by random effects, Conditional = variance explained by fixed and random effects, AIC = Akaike Information Criterion. Italicized text = *p* < 0.05, bolded text = *p* < 0.001

#### Additional analyses

We found a significant main effect of Label on the additional five criteria (*p*’s < 0.001), Emotion, Story, Meaningful, Effort, and Time, such that these criteria were rated higher when the label was “human-created” than “AI-created.” These full models (constructed as the previous Model 1’s with individual-difference measures) are reported in Additional file 1: Table A.

### Study 2 discussion

Study 2 replicated and extended the general anti-AI art sentiments from Study 1. Using more-sophisticated modeling, we again found that participants tended to prefer artworks that were labelled human-created over those labelled AI-created art. Importantly, this pattern of results was observed across all four original criteria, as well as the additional five criteria added in Study 2.

We designed our analyses such that three models predicted scores for each criterion. In Model 1, individual-difference traits were targeted, as they were the only predictor variables inputted besides the Label and Painting Type. In Model 2, we added predictor variables that could vary with each trial—rather than remain static, as with the individual-difference metrics—which were the 5 additional criteria: Emotion, Story, Meaningful, Effort, and Time. Model 2 aimed to identify the contributions that more-communicative criteria could predict over our original four criteria. For instance, how does the ability of an art piece to evoke a story relate to the general liking of that piece, and does this relationship depend on the label? Due to the abundance of predictor variables, several of which were not significant in Model 2, we created a third and final model consisting only of significant main effects and interactions from Model 2, which allowed for easier interpretation and discussion. This tripartite model approach was not specified in the pre-registration, though the basic model type and structure (linear mixed models) were.

As determined by our significant-only model (Model 3), when participants reported Liking some paintings more than others, regardless of Label, we also found an increased ability to extract emotion out of the painting (Emotion), to create a story alongside the painting (Story), to find personal meaning from the painting (Meaningful), and to believe the painting required more effort to produce (Effort). Interestingly, interactions between Label and two criteria (Story, Effort) emerged, but in opposite directions. Specifically, with a greater ability to extract a story from a painting (i.e., higher narrativity), participants liked the painting more if that painting was created by AI as compared to if it was created by humans. Narratives, then, may serve as a catalyst for appreciating products on surface levels. However, if participants believed the painting involved more effort in its creation than other paintings, participants liked paintings made by humans more than those made by AI. This may be due to a lack of understanding, on the participants’ end, of how AI produce pieces, and a need to actually witness the artistic production as investigated in Chamberlain et al. (2018). Additionally, it may reflect an effort heuristic. As suggested by Kruger et al. (2004), people use effort as a metric to inform their judgements of the quality of a product (e.g., poems, paintings, suits of armor; Kruger et al., 2004). These findings suggest this effort heuristic may only exist for human products and not AI products. No individual-difference traits emerged in Liking’s Model 3, but its Model 1 originally found that those with both higher growth-mindset scores and higher fixed-mindset scores liked paintings more in general. That fixed and growth mindsets were both positively associated with aesthetic judgements was surprising, yet interesting. This finding partly mirrors results from Karwowski et al. (2019), who found some people can hold both fixed and growth attitudes toward creativity, and further nuances perceptions of creativity that were formerly considered along a continuum between fixed or growth poles (Karwowski, 2014). The Model 1 also reported a main effect of positive attitudes toward AI, which was qualified with an interaction with the Label, such that with higher appreciation for AI, participants liked AI-labelled art more than human-labelled art.

The final model of Beauty revealed similar main effects and interactions as the final Liking model. There was also an additional main effect of age, such that younger participants found art, regardless of its purported creator, to be more beautiful; this may be in line with findings from Tröndle et al. (2014), who found that younger participants are more willing to ascribe contemporary artistic creations as “art.” An interaction between Label and Openness emerged, such that with higher openness to experience, participants found AI-labelled paintings more beautiful as compared to human-labelled paintings, nuancing claims of openness and aesthetic appreciation by McCrae (2007). No other individual-difference measures emerged in this final model, but in Model 1, we originally had main effects of positive AI attitudes (qualified with an interaction with the Label, similar to the Liking Model 1), and a main effect of fixed mindset. Again, that fixed mindsets emerge is an interesting point that merits future investigation.

We also implemented the same models for our communicative criteria. The final models for Profundity and Worth showed a main effect of Emotion, Story, Meaningful, Effort, and (nonsignificant in Liking and Beauty) Time. For both Profundity and Worth, there was also a significant interaction between the label and positive attitudes toward AI, such that more positive attitudes toward AI led to higher perceived Profundity and Worth when the art was created by AI as compared to by a human. The Profundity final model, but not Worth’s, also had significant effects of growth and fixed mindsets. However, in both Model 1’s of Profundity and Worth, main effects of positive AI attitudes (qualified by an interaction with Label), growth and fixed mindsets, and CRT scores emerged. The main effect specifically suggests that lower CRT scores (i.e., poorer ability to override intuitive responses) led to higher ratings of Profundity and Worth regardless of label. This mirrors work by Pennycook et al. (2015) that investigated profundity and judgements of randomly generated statements, suggesting an overall increased ability to find Profundity and Worth in the environment when having a lowered filter for regulation. Lastly, supplemental analyses (see Additional file 1) also showed that the additional five communicative criteria (Emotion, Story, Meaningful, Effort, Time) behaved similarly in response to an “AI” or “human” label (such that the latter has consistently higher ratings). In other words, a label of “human” acts as a vessel for elaborative and communicative engagement.

Overall, results from Study 2 supported a distinction in judgement patterns between Liking/Beauty and Profundity/Worth across models, replicating and extending these patterns from Study 1. Interestingly, it appears that when raters judge sensory-level criteria, they may rely on some properties that require greater engagement (e.g., story-telling and effort) depending on the label provided. In this regard, it is possible that the sensory-level judgements can be both a consequence of more-passive appraisals based on solely visual features *and* an interaction with other judgement mechanisms that require active engagement with the piece, in line with dual-process models like that of Graf and Landwehr (2015). Meanwhile, when judging communicative criteria like Profundity and Worth, people tend to rate along the lines of other judgements (e.g., time spent on painting), only moderated by personal positive AI attitudes depending on the label. Individual differences, then, may only affect how viewers differently judge AI or human art when judging their communicative properties.

## General discussion

In line with the extant literature on the role of labels in subjective evaluations—both in aesthetics and other domains of preferences (e.g., Kirk et al., 2009; McClure et al., 2004; Newman & Bloom, 2012; Plassmann et al., 2008; Turpin et al., 2019)—our findings demonstrate that labels significantly influence people’s judgements of visual artworks. Specifically, we investigated how a label of “AI-created” on a painting affects the evaluation of the artwork, as compared to a label of “human-created.” In a time of unprecedented growth in AI-art algorithms, websites, and exhibits, it is evermore timely to consider how people perceive, judge, and interact with artwork produced by AI. We found a bias against AI-labelled art across all aesthetic judgement criteria, but more so for appraisals related to art as a deeper communicative medium (Profundity, Worth). In fact, differences in surface-level judgements between human- and AI-labelled art, though influenced by some communicative processes (e.g., Effort and Story), were nearly non-significant. These findings have crucial implications for art, AI, and creativity at large.

Art created by AI has been met with both success and controversy (see Chatterjee, 2022); and, very recently, these creative products have permeated into mainstream culture like museums and artistic industries (Diaz, 2022; Kaleagasi, 2017; Kinsella, 2018; Zulić, 2019). Though contention ensues over whether artificial programs are capable of producing “creative” products (in line with the computer science principle known as the Lovelace Objection; Natale & Henrickson, 2022), it is nonetheless critical to understand how audiences perceive such art given that contemporary psychological literature considers creativity to be not an absolute construct, but a consequence of changing interactions between subjective criteria and socio-cultural factors like technology (i.e., “in the eye of the beholder”; Amabile, 1982; Cseh & Jeffries, 2019; Hennessey et al., 2011; Kaufman & Sternberg, 2010). Probing these attitudes toward AI-art, our studies demonstrate clear preferences for artwork labelled as human-created as compared to AI-created, despite all art being AI-created (which controlled for potential bottom-up discrepancies between actual human- versus AI-created paintings, while also leveraging findings that show AI art is indiscernible from human art; Chamberlain et al., 2018; Gangadharbatla, 2022). However, the degree of preference depended on the type of judgement, as our results show a marked distinction between judgements of Liking/Beauty and Profundity/Worth. In Study 1, for instance, we found increased effect sizes for differences between human and AI labels for the more-communicative criteria of Profundity (*d* = 0.47) and Worth (*d* = 0.61) than Liking (*d* = 0.17) and Beauty (*d* = 0.22). This distinction is further emphasized by the different interaction mechanisms discovered in Study 2. Ultimately, these results align well with hierarchical and multi-processing models of aesthetic encounters that view sensory versus communicative engagements as different pathways, including Graf and Landwehr’s (2015) dual-process model of aesthetic liking and Chatterjee and Vartanian’s (2016) Aesthetic Triad, which delineates a distinction between sensory- and meaning-level systems of aesthetics. Corroborating these models, our results suggest that people may feel cognitively obstructed when engaging deeply with and deriving meaning from artworks that are labelled as created by AI (or have any other label that is pejorative). Equivalently, a “human” label encourages elaborative engagement (e.g., deriving emotion, effort, narratives). However, on quick, surface-level evaluations rather than elaborative appraisals, AI art may be better appreciated.

Several interpretations can be drawn from these results. Firstly, participants may hold a nuanced view of creativity: while humans can achieve creativity across cognition and production, AI can produce only surface-level reflections of creative thinking according to raters. Artworks that are created by humans may reflect a profound human experience—and thus be deemed more monetarily worthy—that AI cannot produce. Indeed, absent the human experience, AI can produce only sensorily similar—still comparatively beautiful and liked—pieces of visual art. This provides a fascinating perspective of future creativity and aesthetics research: one that does not consider creativity as an all-or-nothing trait, but a spectrum of abilities that AI may be able to penetrate.

Even though these two lines of aesthetic engagement emerged, Study 2 showed that sensory-level judgements actually seemed to be influenced in interesting ways by additional judgements and processes. Such moderation effects support sensory versus communicative pathways of engagement with art, but also reflect how they may intertwine. Indeed, this approach of aesthetics reconciles how aesthetic liking has been found to be both a consequence of passive viewing based on solely bottom-up processing of visual features and product of deeper and more-elaborative engagement with the piece (see Graf & Landwehr, 2015). We found support for this dual-process model in Study 2. Specifically, for Liking and Beauty (two sensory judgements), we isolated interactions between the narrativity of a painting and the label, where higher narrativity led to higher Liking and Beauty when the label of the painting was “AI-created” more than when the label was “human-created.” This seems to imply that narrativity is a key element in surface-level evaluations of artwork, especially with non-human artists. This finding also has strong implications for marketing and product branding, as it is reminiscent of the mass production advertising practices in the early twentieth century where consumers were overall hesitant to purchase goods from multinational conglomerates until narratives were paired with the product (Freeman, 2014; Pulizzi, 2012). In other words, imagined backstories helped to overcome biases against machine-made products. This vessel for deeper engagement (and consumerism) provides a way for AI art to, potentially, have higher evaluations outside of simple bottom-up appraisals.

Narrativity has also played key roles in other modes of art. Music has specifically been investigated as a vessel for stories and resulting aesthetic engagements. Such work (e.g., Margulis et al., 2019; McAuley et al., 2021) finds interesting cross-cultural differences, implicating the role of media in our consumption of art and underlying associations (see Wu et al., 2020 for a cross-cultural study that finds higher appreciation for AI products of art from Chinese participants than American participants). The fact that narrativity scores interact with the label of another modality in our study (paintings) strengthens the idea that stories serve as an engine for general art judgement (that is sensitive to context). Future work could further investigate this possibility by experimentally manipulating the level of narrativity of artworks, which would in turn allow for the assessment of the influence of such a manipulation on people’s aesthetic encounters and appraisals. In such a case, we would anticipate that increasing the narrativity associated with a given piece of art would produce more-positive judgement of that art relative to artworks with lower levels of narrativity. Narratives could also be indirectly stimulated; that is, certain titles and terms may automatically evoke stories which may affect ratings, similar to how pseudo-profound randomly generated titles increase perceived profundity for both computer-generated and human-created abstract paintings (Turpin et al., 2019).

Another interaction that emerged in Study 2 was between the perceived effort of paintings and the label, though in the opposite direction as the narrativity interaction: higher perceived effort led to higher Liking and Beauty when the label was “human-created” more than when it was “AI-created.” Kruger et al.’s (2004) “effort heuristic,” then, may only be immediately available for human-made products, and people seek markers of effort in such products. For instance, Chamberlain et al. (2018) found that people observe the meticulous brush strokes (i.e., an/isotropy) in artworks and use these brush strokes as a heuristic for aesthetic judgements. In other words, people may show an appreciation for an artist’s effortfulness (and/or meticulousness) as the labor infuses the product with some temporal or monetary value. That this effort heuristic doesn’t emerge for AI-labelled art could also reflect a general lack of knowledge about how much effort goes into AI-created art (and what “effort” even reflects in the context of AI algorithms). Interestingly, however, Chamberlain et al. (2018) were able to increase appreciation for AI-created art by showing participants videos of anthropomorphized robots creating paintings, which could have reasonably led participants to believe that the AI engaged in greater levels of effort in its artistic process (thereby mitigating the anti-AI bias). Thus, effort heuristics may be probed even in instances of AI-art in future work.

In regard to relationships between judgements and with painting type (abstract versus representational), we only found modest significant interactions between the label and painting type for Liking and Profundity in Study 1, and did not include these interactions in Study 2 as none emerged significantly in initial analyses. This ultimately supports findings from Chamberlain et al. (2018), who found no significant interactions between the label and painting type of general aesthetic preferences. In addition, these findings give nuance to the attribution effects investigated by Gangadharbatla (2022) and Chamberlain et al. (2018), who found an increased willingness to attribute an AI creator to abstract paintings, and a human creator to representational paintings, respectively. It should be emphasized, however, that in the present study, we defined abstract paintings as having partially or completely unrecognizable referents, and representational paintings as having completely recognizable referents. Notably, however, more-strict definitions and criteria to dissociate abstract and representational art could be used in future research. Importantly, by employing stricter criteria for separating abstract from representational art, future research may, unlike in our study, find significant differences across these two painting types. Future work could also explore intermediary categories like partially representational art in such investigations.

Surprisingly, we also found very few significant relationships between our individual-differences measures and our various indices of art judgement. Only in Study 2 did we see robust effects of an individual trait on judgement patterns. That is, personal positive attitudes toward AI interacted with the label such that higher appreciation for AI led to higher appreciation for art labelled as created by AI as compared to human-labelled art; perhaps as we accept and utilize machine learning in a growing technological world, these traits will naturally proliferate among the layperson, leading to growing acceptance of AI art. Overall, though, we were surprised by the lack of other clear predicting individual-difference measures in judgements of art. Though some small effects for age and openness emerged in models of Beauty in Study 2, ultimately, more research is needed to determine what, if any, personal traits predict why one may judge art by AI higher than art by humans across criteria.

### Limitations

Notably, there are some limitations to our studies that should be considered, to appropriately qualify our findings, that will inform improved designs in future research on the topic. Firstly, we cannot be certain of how well we deceived participants with some of our labels (i.e., those that were false in cases wherein the labels indicated that the artworks were created by a human), although we can nevertheless minimize these concerns (at least to some extent) by considering the past literature showing participants’ inability to discriminate between AI- and human-created paintings (Chamberlain et al., 2018; Gangadharbatla, 2022). In any case, moving forward, this concern can be eliminated by explicitly ensuring that participants are not able to accurately determine that all of our stimuli were in fact created by AI. Alternatively, a future study could employ both (actual) AI- and human-created artwork that has been normed on the criteria of note. Secondly, though our judgement criteria provided more nuance to simple “aesthetic pleasure” by asking for an artwork’s Profundity or Worth, for instance, it is possible the very act of asking participants to (perhaps atypically) consider these attributes of an artwork alters their natural evaluation. Third, another consideration is that the label of “AI” is quite broad and therefore loose in its meaning, and this may have differentially influenced aesthetic judgements across participants depending on their understanding of the term; future work could specifically ask participants what “AI” means to them, and have independent raters (who are knowledgeable about AI) rate participant responses as an individual-difference measure of AI knowledge that may moderate and/or mediate results. Relatedly, some participants may know more about the specifics of machine learning than others (including, for example, the amount of effort that goes into producing an AI algorithm, and the amount of time it takes an individual to create an artwork via such an algorithm), which could influence their responses to our various measures. Also, some participants may be inclined to consume potentially biased information about AI through news sources, which could influence their responses. A simple teaching intervention on AI or assessment of past experiences with AI could assuage these concerns.

Lastly, though previous studies have investigated the role of art expertise in judgements of AI versus human aesthetics (and aesthetics in general), here, we did not index rater expertise. However, findings on the effects of expertise have been somewhat mixed. Chamberlain et al. (2018) found no moderating effect of expertise on judgement biases on art created by AI or humans, and Moffat and Kelly (2006) found that non-musicians were surprisingly able to discern AI- versus human-created compositions better than musicians. That said, both Moffat and Kelly (2006) and Darda and Cross (2023) found that the anti-AI bias is actually stronger in the experts, which matches general trends that experts rate harsher (e.g., Lundy & Smith, 2017). However, for experts to have a harsher anti-AI bias than non-experts also goes against claims that “novices are… more non-aesthetically biased in their aesthetic judgments compared with experts,” which suggests that experts are more resistant to label effects (Lundy & Smith, 2017, p. 140). Regardless, research is merited to delineate the true role that background experience has on judgements of aesthetics (including AI aesthetics), though given that experts may have a stronger anti-AI bias as some studies have supported, our results may, if anything, reflect more-conservative effects given our sample was not limited to experts.

## Conclusions

Across two studies, we found evidence of a multi-processing approach to aesthetics, which has strong implications for creative thinking and art industries. Firstly, people preferred (purportedly) human-created art over AI-created art. This preference was particularly evident for criteria that communicated deeper meanings of the art (e.g., Profundity, Worth). On more-sensory levels, the difference between human- and AI-labelled art was much more modest, though significant differences were nonetheless observed. As such, AI-labelled art can still be greatly appreciated (almost as much as human-labelled art) when people utilize varying levels of engagement processes. Interestingly, the sensory-versus communicative-level judgement processes were further distinguished by different interaction processes: the former were moderated by rates of story-telling and perceived effort (though in opposite directions), while the latter were moderated by personal positive attitudes toward AI. These interactions shed light on when and why individuals may appreciate art made by different creators, as posed in our initial research question, and also reflect how different stages of multi-processing models of aesthetics may interact with one another.

In conclusion, people tend to perceive art as reflecting a human-specific experience, though creator labels seem to mediate the ability to derive deeper evaluations from art. Thus, creative products like art may be achieved—according to human raters—by non-human AI models, but only to a limited extent that still protects a valued anthropocentrism.

## Supplementary Information

**Additional file 1:** Additional file 1 includes the Model 1 output for each of the Study 2 additional criteria.

## Data Availability

The datasets supporting the conclusions of this article, and the scripts used to analyze the data, are available on Open Science Framework, and can be found at: https://osf.io/cgw8v/.

## Footnotes

1 Here, we use the term “anti-AI bias.” Of note, however, is that one could reasonably refer to the same bias as a “pro-human bias.” For clarity, and to allow consistency with similar terminology used in previous research on the topic (Chamberlain et al., 2018; Chiarella et al., 2022), we continue to use the term “anti-AI bias.” We thank a reviewer for drawing our attention to this issue of terminology.

2 ANCOVAs controlling for CRT produced the same results, such that images with the “human” label elicited increased ratings than those with an “AI” label. Only for Beauty (as in our difference-score analyses) did CRT moderate this main effect marginally (*F*(1, 147) = 4.200, η^2^ = .004, *p* = .042).

3 The only way to ensure they are not would be to run a norming study; to our knowledge, no such study has yet been run. Future work is therefore merited here.

4 We aimed to recruit one-hundred and fifty participants, as in Study 1 and according to our pre-registration. However, Prolific mistakenly labelled one participant’s task as “unfinished,” thereby leading to the recruitment of an additional participant.

5 Because of the ordinal nature of the outcome data, we did not presume continuous assumptions (e.g., normal residuals) would be met during pre-registration. And, because ordinal data with crossed random effects demand Bayesian models due to the availability of R packages, we thus pre-registered Bayesian models. However, once the data were collected and revealed normal residuals, we could and did run a (frequentist) crossed random effects model. Thus, no Bayesian models were ever run.

## Declarations

### Open practices statement

All data, analysis scripts, stimuli, and the Study 2 pre-registration can be found at https://osf.io/cgw8v/.
