# Supplementary material for: Humans versus AI: whether and why we prefer human-created compared to AI-created artwork
Source: Cogn Res Princ Implic. 2023 Jul 4;8:42. doi: 10.1186/s41235-023-00499-6 (PMC10319694; doi:10.1186/s41235-023-00499-6)
Supplement: Supplementary file 1 — Additional file 1: Additional file 1 includes the Model 1 output for each of the Study 2 additional criteria. [file 41235_2023_499_MOESM1_ESM.docx]

**Supplementary Table A:** Study 2 additional criteria

| **Outcome** | **Emotion** | **Story** | **Meaningful** | **Effort** | **Time (log)** |
| --- | --- | --- | --- | --- | --- |
| **Fixed Effects**  **[95% CI]** |  |  |  |  |  |
| (Intercept) | ***2.06***  ***[1.89, 2.23]*** | ***2.21***  ***[1.99, 2.42]*** | ***1.77***  ***[1.61, 1.93]*** | ***2.03***  ***[1.85, 2.22]*** | ***3.82***  ***[3.38, 4.26]*** |
| Label  (Human = 1) | ***0.27***  ***[0.19, 0.36]*** | ***0.32***  ***[0.25, 0.40]*** | ***0.31***  ***[0.24, 0.38]*** | ***1.23***  ***[1.09, 1.37]*** | ***4.28***  ***[3.89, 4.68]*** |
| Painting Type  (Representational = 1) | ***0.49***  ***[0.33, 0.65]*** | ***0.69***  ***[0.44, 0.94]*** | ***0.47***  ***[0.32, 0.62]*** | ***0.36***  ***[0.18, 0.54]*** | *0.41*  *[0.17, 0.65]* |
| Openness | -0.00  [-0.03, 0.02] | 0.01  [-0.01, 0.03] | -0.01  [-0.03, 0.01] | *-0.03*  *[-0.06, -0.01]* | *-0.08*  *[-0.15, -0.01]* |
| Positive AI | *0.03*  *[0.01, 0.04]* | ***0.03***  ***[0.01, 0.04]*** | *0.02*  *[0.00, 0.04]* | 0.02  [-0.00, 0.03] | -0.03  [-0.08, 0.02] |
| Negative AI | -0.01  [-0.04, 0.01] | 0.00  [-0.02, 0.02] | -0.01  [-0.03, 0.02] | 0.01  [-0.01, 0.04] | *0.09*  *[0.02, 0.16]* |
| Growth | 0.06  [-0.00, 0.12] | 0.03  [-0.02, 0.09] | *0.08*  *[0.02, 0.14]* | *0.10*  *[0.04, 0.17]* | 0.09  [-0.10, 0.28] |
| Fixed | 0.04  [-0.00, 0.08] | 0.03  [-0.01, 0.07] | *0.06*  *[0.02, 0.10]* | *0.06*  *[0.02, 0.11]* | 0.02  [-0.11, 0.15] |
| Empathy | 0.01  [-0.01, 0.03] | 0.01  [-0.01, 0.02] | 0.00  [-0.01, 0.02] | 0.00  [-0.02, 0.02] | 0.02  [-0.03, 0.08] |
| CRT | -0.06  [-0.11, 0.00] | -0.04  [-0.10, 0.01] | *-0.06*  *[-0.12, -0.01]* | -0.06  [-0.12, 0.00] | *-0.23*  *[-0.41, -0.05]* |
| Age | 0.01  [-0.00, 0.02] | 0.01  [-0.00, 0.02] | -0.00  [-0.01, 0.01] | 0.01  [-0.01, 0.02] | 0.01  [-0.03, 0.04] |
| Label ✕ Openness | 0.01  [-0.01, 0.02] | -0.00  [-0.01, 0.01] | 0.01  [-0.00, 0.02] | *0.03*  *[0.01, 0.06]* | 0.06  [-0.01, 0.14] |
| Label ✕ Positive AI | *-0.01*  *[-0.02, -0.00]* | ***-0.02***  ***[-0.03, -0.01]*** | -0.01  [-0.02, 0.00] | -0.01  [-0.03, 0.01] | 0.02  [-0.03, 0.07] |
| Label ✕ Negative AI | -0.01  [-0.03, 0.00] | -0.01  [-0.03, 0.00] | -0.01  [-0.02, 0.00] | -0.01  [-0.03, 0.02] | *-0.08*  *[-0.15, -0.01]* |
| Label ✕ Growth | 0.02  [-0.02, 0.06] | *0.03*  *[0.00, 0.07]* | 0.02  [-0.01, 0.05] | -0.04  [-0.10, 0.03] | -0.02  [-0.20, 0.16] |
| Label ✕ Fixed | 0.00  [-0.02, 0.03] | 0.00  [-0.02, 0.03] | 0.00  [-0.02, 0.03] | -0.03  [-0.08, 0.01] | -0.01  [-0.13, 0.12] |
| Label ✕ Empathy | 0.00  [-0.01, 0.01] | 0.00  [-0.01, 0.01] | -0.00  [-0.01, 0.01] | 0.01  [-0.01, 0.03] | -0.01  [-0.06, 0.04] |
| Label ✕ CRT | -0.03  [-0.07, 0.00] | *-0.04*  *[-0.07, -0.00]* | *-0.04*  *[-0.07, -0.01]* | 0.01  [-0.05, 0.07] | 0.04  [-0.14, 0.21] |
| Label ✕ Age | -0.00  [-0.01, 0.01] | -0.00  [-0.01, 0.01] | 0.00  [-0.01, 0.01] | -0.00  [-0.02, 0.01] | -0.00  [-0.04, 0.03] |
| **Random Effects** |  |  |  |  |  |
| Participant (Intercept) | 0.57 | 0.50 | 0.21 | 0.68 | 6.28 |
| Label (Slope) | 0.15 | 0.09 | 0.06 | 0.67 | 5.76 |
| Painting (Intercept) | 0.04 | 0.12 | 0.04 | 0.06 | 0.10 |
| Residual | 0.80 | 0.91 | 0.53 | 0.60 | 1.47 |
| **Model** |  |  |  |  |  |
| Marginal | 0.13 | 0.14 | 0.58 | 0.31 | 0.45 |
| ICC | 0.44 | 0.40 | 0.32 | 0.51 | 0.77 |
| Conditional | 0.51 | 0.49 | 0.71 | 0.66 | 0.87 |
| AIC | 12101.44 | 12635.76 | 10126.86 | 11095.02 | 15256.81 |

*Note*. CI = confidence interval, Marginal = variance explained by fixed effects, ICC = intraclass correlation or variance explained by random effects, Conditional = variance explained by fixed and random effects, AIC = Akaike Information Criterion. Italicized text = *p* < 0.05, bolded text = *p* < 0.001.
